# Interaction between Persistent Organic Pollutants and ZnO NPs in Synthetic and Natural Waters

**DOI:** 10.3390/nano9030472

**Published:** 2019-03-21

**Authors:** Rizwan Khan, Muhammad Ali Inam, Sarfaraz Khan, Du Ri Park, Ick Tae Yeom

**Affiliations:** 1 Graduate School of Water Resources, Sungkyunkwan University (SKKU) 2066, Suwon 16419, Korea; rizwankhan@skku.edu (R.K.); aliinam@skku.edu (M.A.I.); enfl8709@skku.edu (D.R.P.); 2 Key Laboratory of the Three Gorges Reservoir Region Eco-Environment, State Ministry of Education, Chongqing University, Chongqing 400045, China; Sfk.jadoon@yahoo.com

**Keywords:** agglomeration, interaction, organic pollutants, stability, ZnO nanoparticles

## Abstract

The use of zinc oxide nanoparticles (ZnO NPs) and polybrominated diphenyl ethers (PBDPEs) in different products and applications leads to the likelihood of their co-occurrence in the aquatic system, making it important to study the effect of PBDPEs on the fate and transport of ZnO NPs. In this study, we determine the influence of PBDPEs (BDPE-47 and BDPE-209) on the colloidal stability and physicochemical properties of ZnO NPs in different aqueous matrices. The results indicated the shift in ζ potential of ZnO NP from positive to negative in the presence of both PBDPEs in all tested waters; however, the effect on the NPs surface potential was specific to each water considered. The lower concentration of the PBDPEs (e.g., 0.5 mg/L) significantly reduced the ζ potential and hydrodynamic diameter (HDD) of ZnO NP, even in the presence of high content of dissolved organic matter (DOM) in both freshwater and industrial wastewater. Moreover, both BDPE-47 and BDPE-209 impede the agglomeration of ZnO NP in simple and natural media, even in the presence of monovalent and polyvalent cations. However, the effect of BDPE-47 on the ζ potential, HDD, and agglomeration of ZnO NP was more pronounced than that of BDPE-209 in all tested waters. The results of Fourier transform infrared (FT-IR) and X-ray Photon Spectroscopy (XPS) further confirm the adsorption of PBDPEs onto ZnO NP surface via aromatic ether groups and Br elements. The findings of this study will facilitate a better understanding of the interaction behavior between the ZnO NPs and PBDPEs, which can reduce the exposure risk of aquatic organisms to both pollutants.

## 1. Introduction

Engineered nanoparticles (ENPs) are used in various fields, because of their unique structural properties and high reactivity. Among them, zinc oxide (ZnO) NPs is the third extensively produced ENP, and is used in cosmetics, marine antifouling paints, and packaging. The industrial applications of ZnO include catalysts, photovoltaic devices, lasers, transducers, and sensors [1]. The global production of ZnO NPs was around 1600 tons in 2010, which is estimated to increase to 58,000 tons/year by 2020 [2]. Moreover, a recent study [3] reported the concentration of ZnO NPs in wastewater treatment plant biofilm to be (22–24) µg/Kg, which is anticipated to increase over time. As a consequence, a considerable fraction of ZnO NPs may enter into water, which may pose a threat to aquatic life and human health. Previous studies [4,5] indicated the adverse effects of ZnO NPs on different aquatic organisms, such as zebrafish, sea urchin, algae, bacteria, and plants. A recent study have reported the toxic effect of ZnO NPs on the organisms living on the ground, which include the accumulation of tiny NPs in mice liver causing endoplasmic reticulum (ER) stress response [6]. Accordingly, the effect of ZnO NPs on the reproduction of *Folsomia candida* and *Eisenia* fetida showed that the toxicity of ZnO NPs highly influenced by soil pH, as the rate of dissolution increased in more acidic soils [7,8]. Moreover, the toxic effects of these NPs on human cells, including damage to DNA and cell membranes, have also been well reported [9]. Thus, understanding the fate and mobility of ZnO NPs in water is essential to assess their potential risk in the ecosystem.

The toxicity and colloidal stability of ZnO NPs depend upon several environmental factors, such as primary particle size, pH, ionic strength (IS), dissolved organic matter (DOM), and extracellular polymeric substances (EPS) [10,11,12,13]. The small size of NPs may cause significant variation in the aggregation kinetics under various water matrices. Moreover, they are considered stable in suspension with high capability to convey toxic substance, while large size NPs have a tendency to form large agglomerates which decrease the dissolution of NPs in solution [9]. Some recent studies [14,15] demonstrated that ZnO NPs are unstable in waters with high IS (e.g., seawater); however, high concentration of DOM enhances the colloidal stability. In addition, natural waters may also contain synthetic organic pollutants resulting from direct or indirect anthropogenic activities. Among many persistent organic pollutants (POPs), polybrominated diphenyl ethers (PBDPEs) are mostly used as flame retardants in paints, industrial applications, and other consumer products [16]. Therefore, there are growing concerns regarding the release of PBDPEs into the natural environment during the usage and disposal of these products. Earlier study [17] has shown the adsorption of organic pollutants onto the NPs surface in water. Several developed countries have restricted the use of PBDPEs; however, these pollutants are still found in products manufactured prior to the phase-out completion in these countries. Moreover, their products are frequently available in countries with unrestricted use of PBDPEs, thus leading to their risk in the eco-toxicological context [18]. A few researchers [19,20] have reported the concentration of PBDPEs in surface waters up to 1000 ng/L, while it has been also found in the atmosphere, sediments, and humans [21,22,23]. Moreover, the elevated concentration of organic pollutants such as PBDPEs may lead to their adsorption onto the NPs surface in an aquatic environment, thus enhancing the overall colloidal stability [24]. After an extensive literature search, we found that the interaction between POPs and ZnO NPs in water has barely been touched by the environmental scholars. Moreover, previous studies also seem insufficient regarding the effect of hydrophobic POPs on the environmental fate and mobility of ENPs in water.

Accordingly, the objective of the present study was to investigate the effect of two widely used PBDPEs, BDPE-47 and BDPE-209, on the colloidal stability of ZnO NP in aqueous matrices. Moreover, this work also hopes to assess the influence of POPs on the physicochemical properties of ZnO NPs in both synthetic and natural waters.

## 2. Materials and Methods

### 2.1. Materials and Reagents

The ZnO powder (CAS No:1314-13-2, purity > 97%, see Table S1 of the Supplementary Information (SI) for detailed properties), and two BDPE congeners 2,2′,4,4′-tetrabromodiphenyl ether (BDPE-47) and 2,2′,3,3′,4,4′,5,5′,6,6′-decabromodiphenyl ether (BDPE-209) with purity > 97% were purchased from Sigma Aldrich (St. Louis, MO, USA). Table 1 shows the physicochemical properties of both BDPEs. The potassium chloride (KCl), magnesium chloride (MgCl_2_), aluminum sulfate Al_2_(SO_4_)_3_, hydrochloric acid (HCl), and sodium hydroxide (NaOH) were obtained from Samchun (Samchun Pure Chemicals Co., Ltd., Pyeongteak-si, Korea). The Synergy water system (Milli-Q, Millipore, Burlington, MA, USA) was used to produce deionized (DI) water (18.2 MΩ cm^−1^ resistivity), and it was used in the preparation of the stock solutions.

### 2.2. Preparation of Stock Suspension

The stock solution of ZnO NPs was prepared by adding 100 mg of ZnO powder in 1 L of DI water. The detailed procedure can be found in our previous study [25]. Stock solutions (500 mg/L) of BDPE-47 and BDPE-209 were prepared in anhydrous ethanol and dimethyl sulfoxide (DMSO, purity > 99.5%), respectively, by continuous mixing with a magnetic stirrer for 2 h. The ZnO–PBDPEs suspensions were prepared with a lower ratio of 1:20 (solvent:water) to reduce the effect of solvent during experiments, and further diluted to achieve the desired concentration.

### 2.3. Collection of Natural Waters

To investigate the effect of PBDPEs on ZnO NPs stability in synthetic and natural waters, the freshwater (FW) was prepared in the laboratory, while the industrial wastewater (IWW) was obtained from metal processing industry (Onsan National Industrial Complex, Ulsan, Korea). Table S2 of the SI shows the major properties of both waters. The concentration of various ions present in IWW was measured with ion chromatography (861-Advanced Compact IC, Herisau, Switzerland) using standard methods [26]. The pH and total organic carbon (TOC) of collected samples were measured with pH meter (HACH-HQ40d portable multi meter, Loveland, CO, USA) and TOC analyzer (TOC-5000A, Shimadzu Corporation, Kyoto, Japan). In order to remove the particulates, both waters were filtered using 0.45 µm glass fiber filter.

### 2.4. Effect of PBDPEs on ζ Potential and Size of ZnO NPs

The ENPs stability and their interactions with other molecules in aqueous environment are mostly controlled by the absolute surface charges of the colloids. The controlled experiments were performed to investigate the influence of PBDPEs type (BDPE-47, BDPE-209) and concentration ((0–5) mg/L) on the ζ potential and hydrodynamic diameter (HDD) of ZnO NPs in DI water at pH 7. Moreover, additional experiments were conducted in synthetic and natural waters to quantify the effect of both PBDPEs on the ζ potential and HDD of ZnO NPs in these waters.

### 2.5. Aggregation Kinetics in Various Aqueous Matrix

The aggregation kinetics of ZnO NP was studied through the time-resolved dynamic light scattering (DLS) method. The predetermined amount of ZnO NPs suspension was added into the glass vials with known volume of either DI water or electrolyte solution (KCl, MgCl_2_, and Al_2_(SO_4_)_3_). Afterwards, the vials were instantly vortexed for 5 s, to ensure complete mixing. Subsequently, 1 mL of the suspension was transferred into a DTS0012 cuvette (Malvern) and then placed in the Zetasizer sample chamber for hydrodynamic size measurements. Following the same procedure, further experiments were conducted by adding known concentrations (0–10 mg/L) of BDPE-47 or BDPE-209, prior to the addition of ZnO NPs in solutions. In order to obtain sufficient signal for DLS analysis, the ZnO NPs concentration was set to 10 mg/L. Since the higher polydispersity index (PDI) limits the reliability of particle HDD values, the PDI values above 0.7 in datasets were not considered [29]. The DLS data were collected in triplicates at 30 s intervals for 30 min at 25 °C, while pH was adjusted to 7.0 using 0.1 M HCl or NaOH solution. The aggregation kinetics of ZnO NPs in synthetic and natural waters were determined using Equation (1) as described in earlier studies [30,31]:

(1) $$k_{a}\propto \frac{1}{N_{0}}{\left(\frac{dD_{h}\left(t\right)}{dt}\right)}_{t\to 0}\to k_{a}.N_{0}={\left(\frac{dD_{h}\left(t\right)}{dt}\right)}_{t\to 0}$$

where *k_a_* is the aggregation rate constant, and *N*_0_ is the initial particle number concentration. The early stage aggregation period was defined for *D_h_* values equal to, or less than two times of initial *D_h_* [30].

The attachment efficiency α was used to measure ZnO NP aggregation kinetics in various waters [32]. The α can be determined from the aggregation rate constant (*k_a_*) normalized by the aggregation rate constant (*k_a_*)*_fast_* in the diffusion-limited regime, as shown in Equation (2):

(2) $$\alpha =\frac{1}{w}=\frac{k_{a}}{k_{a\mathrm{fast}}}={\frac{\frac{1}{N_{0}}{\left(\frac{dD_{h}\left(t\right)}{dt}\right)}_{t\to 0}}{{\frac{1}{\left(N_{0}\right)}}_{\mathrm{fast}}\left(\frac{dD_{h}\left(t\right)}{dt}\right)}}_{t\to 0,\;\mathrm{fast}}$$

where (*k_a_*)*_fast_* shows the favorable suspension condition, where fast, diffusion-limited aggregation occurs [33].

Furthermore, the intersection of two lines extrapolated through the diffusion-limited and reaction-limited regimes yields the critical coagulation concentration (CCC) of KCl for ZnO NP [34]. The CCC can be defined as the minimum amount of an electrolyte required to destabilize NP suspension completely. The value of CCC provides essential information about NPs stability, and can thus be used to predict the fate and transport of NPs in natural waters [14,23].

### 2.6. Other Analytical Procedures

The ζ potential of ZnO NPs was measured with Zetasizer (Nano ZS90, Malvern Instruments, Worcestershire, UK). The instrument was equipped with a 633-nm red laser and was capable of analyzing particles with diameters ranging from 0.3 nm to 5.0 micron using DLS as the basic principle of operation, and ζ potential measurement through Doppler electrophoresis. Folded capillary cell (DTS1060) was used for ζ potential measurements, and disposable low volume polystyrene (DTS0012) cuvette was used for particle size measurement. The ζ potential (mV) was measured at 25 °C with 10 repeated measurements, where the refractive indices of ZnO and water were set to 2.00 and 1.33, respectively. The absorption spectra of ZnO NPs were determined using UV–Vis spectrophotometer (Optizen-2120, Mecasys, Daejeon, Korea) in the 250–800 nm wavelength range. The X-ray photoelectron spectroscopy (XPS), Raman spectroscopy, X-ray diffractometry (XRD), and Brunauer–Emmett–Teller (BET) surface area analysis of the ZnO NPs were done using XSAM HS (KRATOS), D max C III (Rigaku Corporation, Tokyo, Japan), Jobin Yvon microscope (Horiba, Bensheim, Germany), and ASAP 2020 (Micromeritics, Norcross, GA, USA) respectively. Fourier transform infrared (FT-IR) spectroscopy (JASCO, FT-IR-4700, Easton, PA, USA) of ZnO and PBDPEs before and after interaction were conducted to explore the possible attachment of functional groups during the interaction between the two pollutants. Moreover, XPS analysis of ZnO–PBDPEs complexes was conducted to confirm the presence of different elements in the structure of ZnO NP after interaction with PBDPEs.

## 3. Results

### 3.1. ZnO NP Characterization

**Figure 1:**
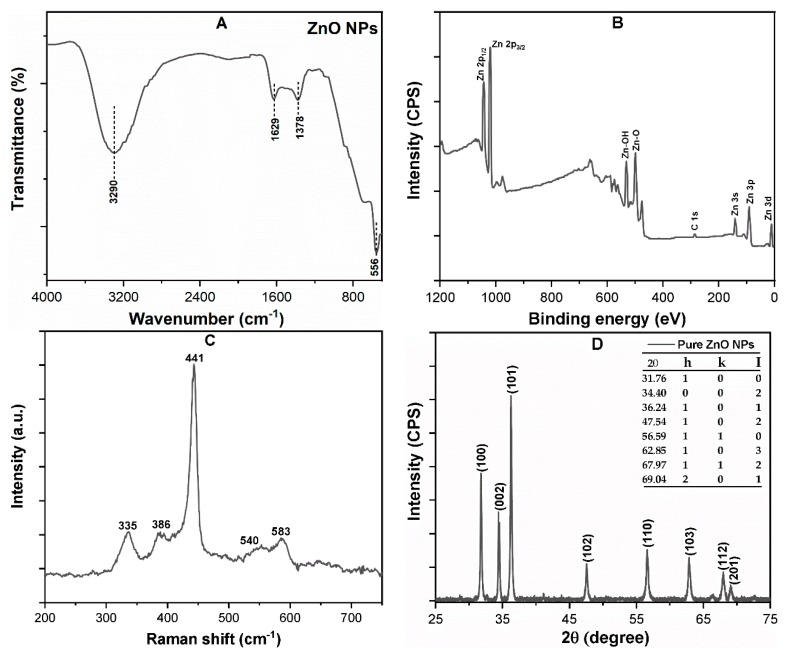
Characterization of the zinc oxide nanoparticles (ZnO NPs) powder (**A**) FT-IR; (**B**) X-ray photoelectron spectroscopy (XPS) survey spectrum; (**C**) Raman spectrum; (**D**) XRD spectra expressed in arbitrary units and counts per second.

Figure 1A shows the FT-IR spectroscopy of pristine ZnO powder, which reveals the broad band at 556 cm^−1^ corresponding to the stretching vibration of Zn–O bonds [25]. Moreover, the peaks observed at 1378, 1629, and 3402 cm^−1^ are attributed to the OH groups and displacement of weakly adsorbed water molecules on the NPs surface. Figure 1B shows the XPS spectra of pure ZnO, which revealed that the corresponding binding energies of Zn 2p_1/2_ and 2p_3/2_ were 1042 and 1021 eV, respectively [35]. In addition, the Raman scattering showed peaks at 386 and 441 cm^−1^, which correspond to A1 (TO) and E1 (TO) mode (Figure 1C).

The XRD spectrum pattern confirms the crystalline structure of the ZnO NPs (Figure 1D). In the spectrum, peaks observed at 31.76°, 34.37°, 36.24°, 47.54°, 56.58°, 62.85°, and 67.97°, were corresponding to (100), (002), (101), (102), (110), (103), and (112) planes of pure ZnO, respectively. Therefore, all detectable peaks can be indexed to the ZnO wurtzite structure (JCPDS: 00-036-1451) [36]. In addition, the crystallite size of ZnO NPs was determined using Scherer formula [37], and the average size of ZnO NPs has been calculated to be 45 ± 2 nm. The detailed description can be found in (Supplementary Materials, Section 1.1). Furthermore, Figure S1A of the SI shows the Brunauer–Emmett–Teller (BET) surface area of the ZnO NPs to be around 12.5 m^2^/g.

### 3.2. Effects of PBDPEs on the ζ Potential of ZnO NPs

**Figure 2:**
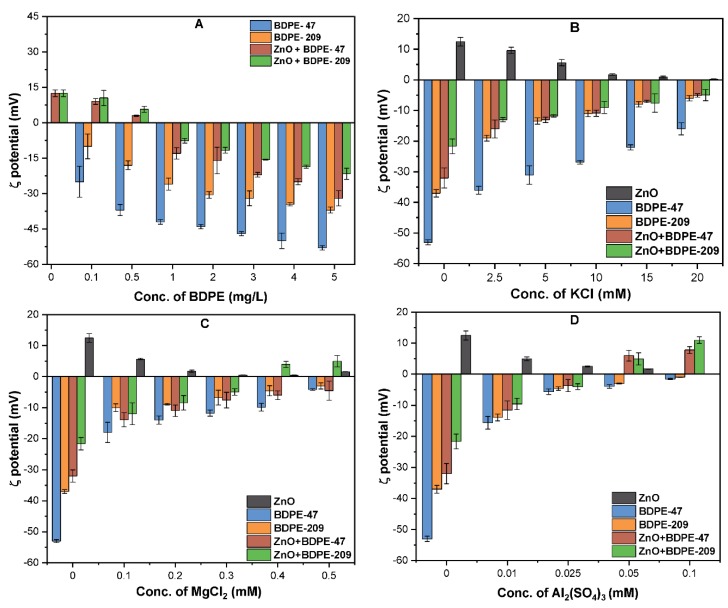
(**A**) ζ potential of ZnO NPs with polybrominated diphenyl ethers (PBDPEs) ((0–5) mg/L) in DI water. ζ potential of BDPEs and ZnO NPs coated with (5 mg/L) PBDPEs in the presence of (**B**) KCl; (**C**) MgCl_2_, and (**D**) Al_2_(SO_4_)_3_ electrolytes.

Figure 2 presents the effects of both PBDPEs on the ζ potential of ZnO NPs (10 mg/L) at pH 7 in the absence and presence of the electrolyte. As indicated in Figure S1B of the Supplementary Information, the ζ potential of ZnO NPs was +12.6 mV, while the isoelectric point (pH_iep_) was observed to be approximately 9.2, which is consistent with previous studies [38,39]. The ζ potential of ZnO NPs became negative upon the addition of BDPE-47 and BDPE-209 (Figure 2A). It is noteworthy that the higher concentration of both PBDPEs resulted in a further decrease in ζ potential (more negative) of the ZnO NPs. For example, the significant decrease in surface charge of ZnO NPs in the presence of 5 mg/L of BDPE-47 and BDPE-209 was found to be −33.2 and −27.1 mV, respectively. However, the ζ potential of 5 mg/L BDPE-47 and BDPE-209 in DI water was observed to be −50.0 and −42.8 mV, respectively (Figure 2A).

These results suggest that both PBDPEs interact with ZnO NPs and reverse their surface potential from positive to negative, thus enhancing the overall colloidal stability [28]. In addition, the adsorption of both PBDPEs on the ZnO NPs surface might decrease the van der Wall (vdW) forces, and increase steric hindrance among the NPs [23]. A more prominent effect of BDPE-47 on the ζ potential was observed as compared to BDPE-209, which might be ascribed to greater electrostatic repulsion and more steric stabilization.

The effects of various electrolytes ((0–20 mM) KCl, (0–0.5 mM) MgCl_2_, and (0–0.1 mM) Al_2_(SO_4_)_3_) on the ζ potential of ZnO NPs in the presence of both PBDPEs (5 mg/L) were investigated (Figure 2B–D). The presence of all electrolytes significantly increases the ζ potential of ZnO NPs in the presence of BDPE-47 and BDPE-209. For example, the ζ potential of NPs with BDPE-47 indicated significant shifts to be −5.0, −4.5, and +7.7 mV at the concentration of each electrolyte, i.e., KCl (20 mM), MgCl_2_ (0.5 mM), and Al_2_(SO_4_)_3_ (0.1 mM), respectively. The effect of trivalent cations on the ζ potential of ZnO NPs was more pronounced, as it required 0.1 mM Al_2_(SO_4_)_3_ to reverse the surface charge from (−52.0 to +7.7) mV with BDPE-47. The possible explanation for such phenomena may be related to the effective charge screening, due to the adsorption of the cations around the EDL of the NPs [10,13]. Moreover, according to the Schulze–Hardy rule, higher valence cations, such as Mg^2+^ and Al^3+^, have a greater ability for charge screening. These results are consistent with a previous studies [40,41], which reported that trivalent cations effectively compress the EDL of NPs in the solution. Furthermore, the ζ potential of BDPE-47 and BDPE-209 indicated a similar trend in the presence of each electrolyte (Figure 2B–D). Our results are in good agreement with earlier studies [14,36] that found charge screening and the neutralization effect from counterions played a substantial role, in comparison to the compressive effect of co-ions.

### 3.3. Effects of PBDPEs on the HDD of ZnO NPs

**Figure 3:**
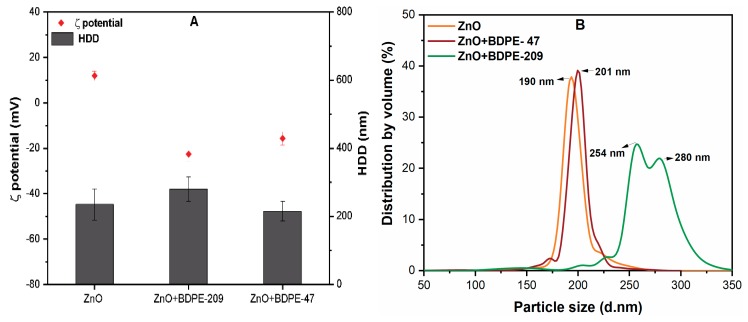
(**A**) Hydrodynamic diameter (HDD) of ZnO NPs (10 mg/L) with and without PBDPEs (5 mg/L) in DI water; (**B**) distribution by volume (%) of ZnO NPs in the presence of (5 mg/L) of BDPE-47 and BDPE-209 at pH 7.

Figure 3 shows the effects of (5 mg/L) BDPE-47 and BDPE-209 on the HDD and size distribution of ZnO NPs suspension. Prior to HDD measurement, the influence of solvent on the ZnO NPs aggregation was determined, which showed that the aggregation kinetics remains the same in the absence and presence of solvents (Figure S1C of the SI). The ZnO NP suspension obtained in DI water showed intensity-weighted HDD of 226 nm, which was much larger than the vender-reported primary particle size (<50 nm). Such observation may be attributed to enhanced vdW forces among the NPs, thereby forming large NP aggregates in aqueous solution [14]. The HDD of ZnO NPs increased to 278 nm in the presence of BDPE-209; however, HDD remained the same (~235 nm) in the case of BDPE-47 (Figure 3A). Moreover, the size distribution of ZnO NPs in DI water and BDPE-47 showed that NPs typically exist in the range of 180–250 nm, while in the case of BDPE-209, the size distribution was found to be between 250 and 350 nm (Figure 3B). These results indicate that the size distribution of NPs in suspension highly depends upon the aggregation, as well as the magnitude, of the surface coating of PBDPEs [29]. It is noteworthy that HDD and the size distribution of ZnO NPs slightly increase in the presence of BDPE-209, which might be ascribed to its higher molecular weight, as compared to BDPE-47 (Table 1). In addition, the enhancement in size distribution of ZnO NPs in the presence of BDPE-209 as compared to BDPE-47 and DI water may also suggest the lower colloidal stability of ZnO NPs in suspension.

### 3.4. Effects of PBDPEs on ζ Potential and HDD of ZnO NP in Natural Waters

**Figure 4:**
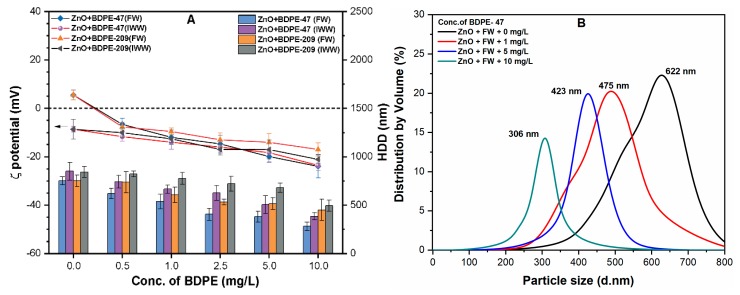
(**A**) The ζ potential and HDD of ZnO NPs coated with different concentrations (0–10 mg/L) of both BDPEs in freshwater (FW) and industrial wastewater (IWW); (**B**) size distribution of ZnO NPs with various BDPE-47 (0, 1, 5, and 10 mg/L) concentrations in FW.

The ζ potential of ZnO NP in FW and IWW was found to be 5.51 and −8.64 mV, respectively (Figure 4A). The interactions between NPs and organic matter present in IWW resulted in the charge reversal of ZnO NPs; moreover, the cations present in FW provide an effective EDL compression on the NPs surface [42]. The addition of low concentration (0.5 mg/L) of both PBDPEs in these waters reduces the ζ potential of ZnO NP with different degrees (Figure 4A). It can be observed that the ζ potential of ZnO NP in FW reduces from 5.51 to −6.8 mV in the presence of 0.5 mg/L of BDPE-47, while it further decreases upon increasing the BDPE-47 concentration. For example, at higher concentration (10 mg/L) of BDPE-47 and BDPE-209 in FW, the reduction in surface charge of ZnO NPs was observed to be −18.5 and −22.0 mV, respectively. Moreover, the results of ζ potential of ZnO NP in IWW showed a similar trend, which was indicated (−18.0 and −17.4 mV) in the presence of 10 mg/L of BDPE-47 and BDPE-209, respectively. It is noteworthy that the effect of PBDPEs on the ζ potential of ZnO NP in FW was more pronounced than that of IWW.

Figure 4A shows significant increases in the HDD of ZnO NPs from 226 to 659 nm in FW and 769 nm in IWW. Moreover, in comparison to DI water, the particle size of ZnO NPs was found to be in the range of 400–850 nm in FW, and 350–900 nm in IWW (Figures S2A, S3A, and S4A of the Supplementary Information). This might be attributed to the higher conductivity in FW (150 µS/cm) and IWW (619 µS/cm), which might result in the formation of large agglomerates. The reduction in HDD and size distribution was observed upon the addition of both PBDPEs for all tested concentrations (Figure 4A,B and Figures S2–S4).

For example, the addition of 0.5 mg/L of BDPE-47 reduces the HDD of NPs to 306 nm (FW) and 493 nm (IWW), which further decrease upon increasing BDPE-47 concentration in these waters. The higher concentration (10 mg/L) of BDPE-47 and BDPE-209 presented low HDD values of 280 and 385 nm in FW, and 450 and 495 nm in IWW, respectively. This is consistent with our previous ζ potential observation, which indicated the significant influence of both PBDPEs, even at the high concentration of DOM present in natural waters (Table S2). It is interesting to note that the more drastic decrease in HDD of ZnO NPs was observed with the increasing concentration of BDPE-47 in comparison to BDPE-209. The shifts in size range further specify that the smaller size NPs may form due to the coating of PBDPEs onto ZnO NPs surface in natural waters, which may enhance the bioavailability and toxicity to the aquatic environment. Therefore, aggregation kinetic studies were conducted in the presence of various electrolytes to understand the fate and transport of the NPs in the heterogeneous aqueous environment.

### 3.5. Effects of PBDPEs on the Aggregation Kinetics of ZnO NP

**Figure 5:**
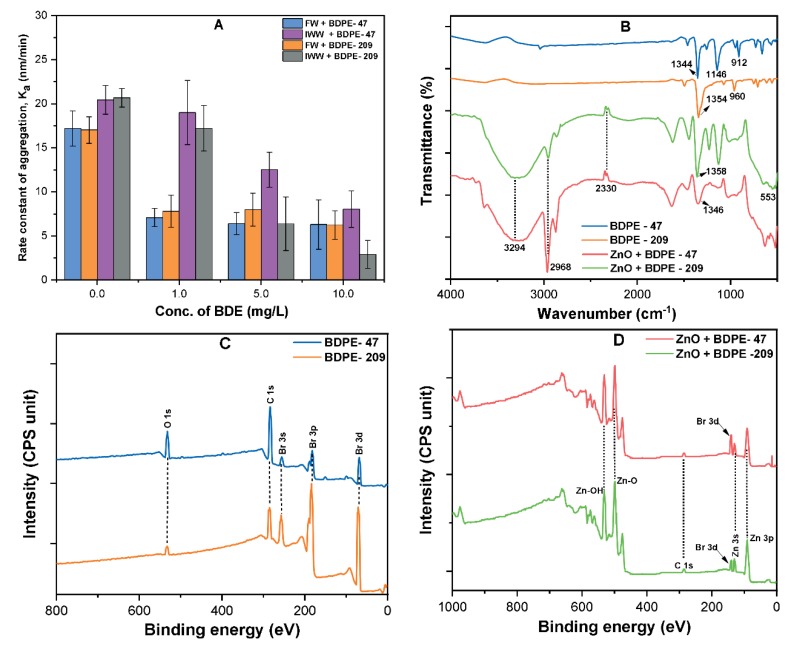
(**A**) Aggregation rate constant (*k_a_*) of ZnO NPs at various concentrations (0–10 mg/L) of both BDPEs in FW and IWW; (**B**) FT-IR spectra of both pristine BDPEs and ZnO NPs coated with BDPE-47 and BDPE-209; (**C**) XPS survey spectra of both BDPEs; and (**D**) ZnO NPs coated with BDPE-47 and BDPE-209.

Figure 5A shows the effects of BDPE-47 and BDPE-209 on the aggregation kinetics (30 min) of ZnO NPs in the presence of 20 mM KCl in both waters that were studied. It can be observed that the NPs suspension become unstable in the absence of PBDPEs, as indicated by the higher HDD value that reached up to 600 nm within the studied period (Figure S5A,B). The addition of 0.1 mg/L BDPE-47 slightly suppressed the aggregation, thereby reducing the HDD by up to ~500 nm (Figure S5A). Furthermore, the increase in BDPE-47 concentration to 5 mg/L remarkably suppressed the aggregation, as well as decreasing the HDD value (220 nm). In contrast, the effect of BDPE-209 on the aggregation of NPs was found to be insignificant where HDD of ZnO NPs observed ~600 nm, even at 5 mg/L of BDPE-209 (Figure S5B). Interestingly, the aggregation of NPs was fully suppressed at higher BDPE-209 concentration (10 mg/L), as shown in Figure S5C. These results suggest the higher stabilizing ability of BDPE-47 than BDPE-209, even at low concentration. This phenomenon is in good agreement with our previous observation that the higher adsorption of BDPE-47 molecules onto ZnO NPs contributes to higher surface charge than for BDPE-209.

To further understand the influence of PBDPEs on the colloidal stability, the aggregation rates (*k_a_*) were measured using the kinetic data. In the absence of PBDPEs, the *k_a_* value reached to 13.75 nm/min, while it reduced to 0.87 nm/min at the concentration of 5 mg/L BDPE-47 (Figure S5D). In contrast, the effect of BDPE-209 on the stability of ZnO NPs was insignificant, even at higher concentration (5 mg/L). Moreover, a slight decrease in the aggregation rate of ZnO NPs was observed, where the *k_a_* was found to be 10.38 and 9.96 nm/min upon the addition of 0.5 and 1 mg/L BDPE-209, respectively (Figure S5D). This observation indicated that the sorption of PBDPEs onto NPs surface mainly occurs via electrostatic forces, thereby affecting the pH and IS of the solution [15]. Another possible reason might be related to the competitive adsorption behavior between cations and PBDPEs onto the NPs surface, which increases with the hydrophobicity of the organic pollutant. It is therefore noteworthy that the influence of 20 mM KCl on the binding affinity of ZnO NPs was maximum in the presence of BDPE-209 (for concentration up to 5 mg/L), rather than for the less hydrophobic BDPE-47.

Figure 5A shows the results of the aggregation kinetics and rates (*k_a_*) of ZnO NPs in the absence and presence of PBDPEs in natural waters. It can be observed that the addition of PBDPEs in natural waters resulted in the decrease in *k_a_* values (Figure 5A). The aggregation rate of ZnO NPs in FW significantly decreased from 17.5 to 7.5 nm/min upon the addition of 1 mg/L BDPE-47; afterwards it slightly decreased to 6.8 nm/min at a higher concentration of BDPE-47 (10 mg/L). Similar trends have been observed in FW containing BDPE-209; however, both PBDPEs showed discrepant aggregation kinetics behavior in IWW (Figure S6). These results indicate that the lower concentration of PBDPEs may significantly influence the fate and mobility of ZnO NPs, even in waters containing high DOM concentration. The low solubility (1–30 µg/L) of organic pollutants in water might enhance the stability of ZnO NPs, thereby reducing the risk of releasing Zn^2+^ ions into natural water bodies. Moreover, the extremely high hydrophobicity of BDPE-209 (log K_ow_ = (6.27–9.97)) and BDPE-47 (log K_ow_ = (6.77–6.81)) might facilitate the adsorption of organic pollutant onto ZnO NPs surface in these waters [14,23]. These results strongly suggest that a low concentration of PBDPEs and other organic contaminants may adsorb on the NPs surface via hydrophobic ligands, thereby affecting the overall colloidal stability.

The interaction behavior of PBDPEs with ZnO NPs depends upon the surface charges, physicochemical interactions, and characteristics of PBDPEs. Moreover, vdW forces and hydrogen bonding might play a vital role during the interactions between NPs and PBDPEs [39]. Figure 5B shows the FT-IR spectra of ZnO NPs before and after interactions with BDPE-47 and BDPE-209. The peaks at ~3294, ~2968, and ~2330 cm^−1^ that appear in ZnO–PBDPEs complex correspond to the stretching vibrations of OH, and symmetric stretching vibration of aliphatic C–H and C=O, respectively [19,25]. In comparison to the pristine ZnO NPs, a new band was observed at ~1346–1358 cm^−1^, which is attributed to the adsorption of PBDPEs onto ZnO NPs surface via aromatic ether (-O-) groups [27,42] Furthermore, Figure 5C,D, indicate the XPS analysis of PBDPEs before and after the interaction with ZnO NPs, which further confirm the attachment of Br (present in BDPE-47 and BDPE-209) onto the ZnO NPs surface [43,44] Therefore, it can be inferred that surface Zn-OH groups (donor) of the NP and ether (-O-) groups in PBDPEs (acceptor), or induced dipole, might be responsible for the strong interaction between PBDPEs and ZnO NPs.

### 3.6. Effects of PBDPEs on ZnO Colloidal Stability

**Figure 6:**
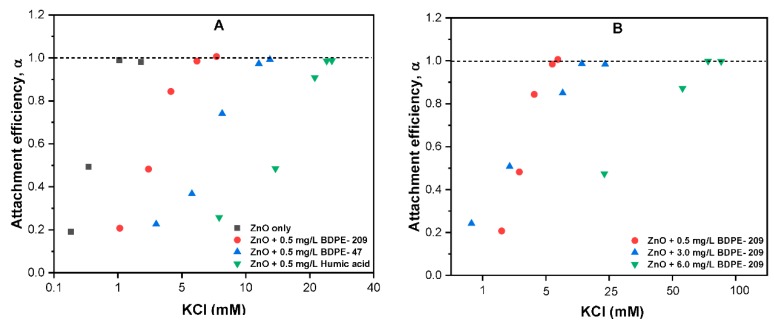
(**A**) Effects of 0.5 mg/L BDPE 47, BDPE 209, and humic acid (HA) on the critical coagulation concentration (CCC) of KCl, and (**B**) effects of different concentrations of BDPE-209 on the CCC of KCl for ZnO NPs (10 mg/L) at pH 7.

The stability of ZnO NPs was further explored by measuring the CCC of KCl for ZnO NPs in the absence and presence of PBDPEs in aqueous media (Figure 6). The energy barrier among the particles is diminished at CCC and electrolyte concentrations above CCC, and hence promotes the diffusion-controlled aggregation. The commonly used DOM substitute humic acid (HA) was used to compare the NPs stability with PBDPEs. Different diffusion-limited agglomeration (DLA) and reaction-limited agglomeration (RLA) regimes were found in the absence and presence of both PBDPEs (Figure 6). The CCC of KCl for ZnO NPs in the absence of PBDPEs was found to be 1.3 mM. This has practical implications in natural waters with IS (1–15 mM), suggesting the rapid settling of ZnO NPs in those environments [10,14]. Moreover, increasing IS weakens the electrostatic repulsion forces and increase the attractive forces among the NPs, thereby enhance the sedimentation due to an increase in attachment efficiency [34,45]. Upon the addition of 0.5 mg/L BDPE-47 and BDPE-209, the CCC of ZnO NPs increase to 6.8 and 11.7 mM KCl, respectively, which indicate the enhancement in stability of ZnO NPs in the presence of PBDPEs (even at low concentrations). This may be attributable to the surface coating of organic pollutant onto NPs increasing the steric hindrance, thus reducing the compression-induced effect of electrolytes [46]. Compared with organic pollutants, the HA increases the CCC of KCl for ZnO NPs two-fold, i.e., 32 mM. These results are in good agreement with previous [13,36,39] that affirm that high molecular weight (HMW) compounds such as HA have higher affinity of sorption onto NPs, due to the presence of several aliphatic and aromatic groups. These results are also consistent with our previous DLS observation, where BDPE-209 shows an insignificant effect on the aggregation of NPs for concentration up to 5 mg/L (Figure S5). Thus, further experiments were conducted to quantify the effect of different concentration of BDPE-209 on the CCC of KCl for ZnO NPs (Figure 6B). The slight increase in CCC was observed to be 20 mM KCl at 3 mg/L BDPE-209 concentration; the CCC becomes 78 mM when the BDPE-209 concentration reaches 6 mg/L. Similar behavior has been observed in the kinetic aggregation study, where the stability of ZnO NPs was significantly increased above 5 mg/L BDPE-209 concentration (Figure S5C). In general, such observations suggest that PBDPEs might improve the colloidal stability of ZnO NPs in the heterogeneous aqueous environment.

### 3.7. Effects of Electrolyte Type on Aggregation Kinetics

**Figure 7:**
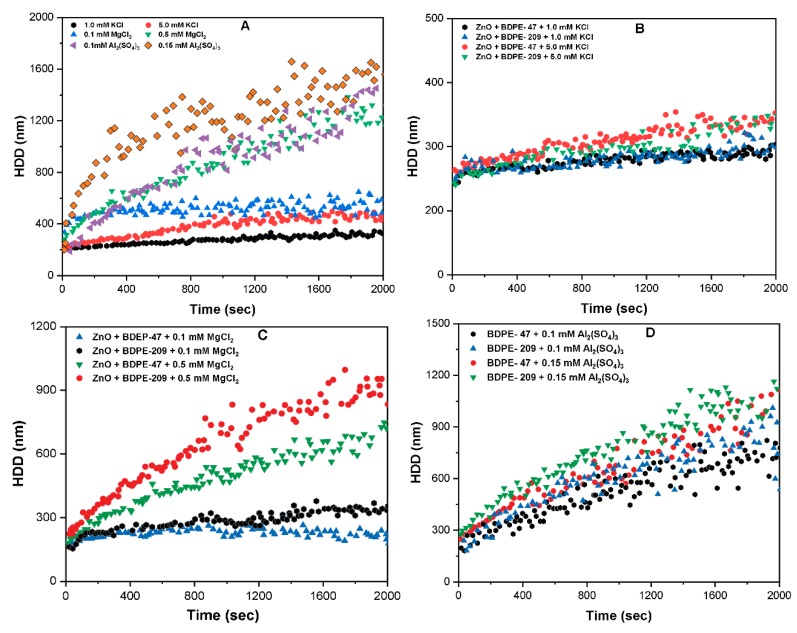
Influence of BDPE-47 and BDPE-209 on the aggregation kinetics of ZnO NPs (10 mg/L) in (**A**) KCl, MgCl_2_, and Al_2_(SO_4_)_3_ at different IS ((0–5) mM); (**B**) monovalent (0–5mM) KCl; (**C**) divalent ions ((0.1 and 0.5) mM of MgCl_2_); and (**D**) trivalent ions ((0.1 and 0.15) mM Al_2_(SO_4_)_3_) at pH 7.

To further understand the effect on the aggregation behavior of ZnO NPs in the presence of various cations and PBDPEs, additional experiments were performed (Figure 7). The aggregation kinetics and rate (*k_a_*) of ZnO NPs at 1 and 5 mM KCl were found to be 320 and 430 nm, and 7.2 and 12.9 nm/min, respectively (Figure 7A and Figure S6). This might be related to the closeness to the value of CCC of ZnO NPs for KCl; thus, most of the NPs are unstable under these conditions. Similar observation was reported in an earlier study [23,39], where enhanced agglomeration was observed in the presence of K^+^, as depicted by the larger HDD of NPs. In contrast, lower concentration of MgCl_2_ (0.5 mM), Al_2_(SO_4_)_3_ (0.15 mM) significantly destabilized the NPs in solution, as described by their higher HDD (1250, 1600 nm) and *k_a_* (89.3, 92 nm/min) values (Figure 7A and Figure S6B,C). These results are in good agreement with the recent study [10,38], that demonstrated the greater charge screening ability of polyvalent cations, thus effectively destabilizing the NPs in suspension.

The addition of PBDPEs suppresses the ZnO NPs agglomeration even in the presence of electrolytes, regardless of their type and concentration (Figure 7B,D). For example, the addition of 0.5 mg/L of BDPE-47 and BDPE-209 in ZnO NPs suspension containing 5 mM KCl decreases the HDD to 350 and 370 nm (Figure 7B). Moreover, the value of *k_a_* of ZnO NPs decreases to 5 and 7 nm/min in the presence of both PBDPEs (Figure S7A). The greater decrease in *k_a_* value in the presence of BDPE-47 may be attributable to the higher stabilizing ability, as observed in our previous section results. The addition of BDPE-209 and BDPE-47in ZnO NPs suspension containing divalent cations (0.5 mM MgCl_2_) slightly decreased the HDD of 900 and 700 nm and *k_a_* of 71 and 54 nm/min, respectively (Figure 7C and Figure S7B). However, the effect of BDPE-47 and BDPE-209 in ZnO NPs suspension with trivalent cations (0.15 mM Al_2_(SO_4_)_3_) on the colloidal stability of NPs was insignificant (Figure 7D and Figure S7C). In general, the stabilization ability of PBDPEs showed a remarkable effect in the presence of monovalent cations, as compared to polyvalent cations. This might be related to the competitive adsorption, as well as the strong interactive behavior between the contaminants in the ternary environment. However, further study is needed to confirm this interpretation.

### 3.8. Study Significance

This study is the first approach to provide some insight into the interaction behavior of ZnO NPs in synthetic and natural waters containing different PBDPEs. Several studies study [13,15,25,34], have described the influence of DOMs on the individual parameters of ENPs. Although the PBDPEs found in the natural environment are at very low concentration, due to their hydrophobic characteristics, they are mostly found in the dissolved state. Thus, the large surface area of ZnO NPs may provide active absorption site to these hydrophobic compounds, and could alter their fate and transport behavior in natural water bodies. The higher concentration of PBDPEs was used in the present short-term study to simulate the amount of POPs absorbed onto the ZnO NPs surface for a more extended period. Moreover, such concentration of PBDPEs (i.e., 0.5 mg/L) facilitates an understanding of the possible interaction mechanism of PBDPEs with ZnO NPs.

## 4. Conclusions

In the present work, we investigated the interaction behavior between ZnO NPs and the two common organic pollutants, BDPE-47 and BDPE-209, in synthetic and natural waters. The results showed that at similar concentrations, BDPE-47 improved the stability of ZnO NPs suspension more than did BDPE-209. Moreover, the BDPE-47 imparts a higher surface potential and more effective surface coating of the ZnO NP, than does BDPE-209. The presence of both PBDPEs suppresses the aggregation rate of ZnO due to the electrosteric hinderance effect, even in the presence of monovalent and polyvalent cations. The FT-IR analysis of ZnO–PBDPEs complexes indicated that aromatic ether groups of PBDPEs played an essential role in the interactions between the POPs and NPs. The results of XPS further confirm the attachment of Br onto the ZnO NPs surface. The presence of both PBDPEs in environmental waters (freshwater and industrial wastewater) results in a discrete adverse effect on the aggregate kinetics and rate of ZnO NPs. Further research and endeavors will focus on the impact of other factors, such as media pH, DOM type, etc., on the interaction behavior between PBDPEs and ZnO NPs in the aquatic system. The findings of this study provide new insights into the enhanced stability of PBDPEs-sorbed ZnO NPs in natural bodies, which may influence the fate and mobility of NPs, thereby increasing their exposure risk to aquatic life and humans.

## Figures and Tables

**Table 1:** Important properties of the PBDPEs used in the present work.

Property	BDPE-209	BDPE-47
Solubility (µg/L)	20–30	1–15
Log k_ow_	6.27–9.98	6.77–6.81
Molecular weight (g/mol)	959.2	485.79
Empirical formula	C_12_Br_10_O	C_12_H_6_Br_4_O
Molecular structure	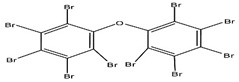	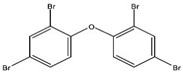

Sources: [22,27,28].

## Supplementary Materials

The following are available online at https://www.mdpi.com/2079-4991/9/3/472/s1, **Figure S1**. (A) Brunauer–Emmett–Teller (BET) analysis of zinc oxide nanoparticles (ZnO NPs) powder; (B) isoelectric point of ZnO NPs in DI water; (C) aggregation kinetics of ZnO NPs in the absence and presence of both solvents used for dissolving the polybrominated diphenyl ethers (PBDPEs); and (D) hydrodynamic size distribution of ZnO NPs in DI water after 30 min sonication. **Figure S2**. Volume distribution of ZnO NPs (10 mg/L) with different concentrations of BDPE-47 of (A) 0 mg/L; (B) 1 mg/L; (C) 5 mg/L; and (D) 10 mg/L in in industrial wastewater (IWW). **Figure S3**. Volume distribution of ZnO NPs (10 mg/L) with different concentration of BDPE-209 of (A) 0 mg/L; (B) 1 mg/L; (C) 5 mg/L; and (D) 10 mg/L in freshwater (FW). **Figure S4**. Volume distribution of ZnO NPs (10 mg/L) with different concentration of BDPE-209 showing (A) 0 mg/L; (B) 1 mg/L; (C) 5 mg/L; and (D) 10 mg/L in IWW. **Figure S5**. Effects of different concentrations (0–5 mg/L) of (A) BDPE-47 and (B) BDPE- 209 on the aggregation kinetics of ZnO NPs (10 mg/L) in the presence of 20 mM KCl at pH 7; (C) aggregation kinetics of ZnO NPs in DI water containing 0, 5, and 10 mg/L BDPE-209, while inset showing aggregation rates at same concentrations; and (D) aggregation rates of ZnO NPs at various concentrations (0–5 mg/L) of BDPE-47 and BDPE-209. **Figure S6**. Effects of (A) BDPE-47 and (B) BDPE-209 on the aggregation kinetics of ZnO NPs (10 mg/L) in FW, and (C) BDPE-47 and (D) BDPE-209 on the aggregation kinetics of ZnO NPs in IWW. **Figure S7**. Aggregation rates of ZnO NPs (10 mg/L) in the absence and presence (1 mg/L) of BDPE-47 and BDPE-209 with (A) monovalent (KCl); (B) divalent (MgCl_2_); and (C) trivalent Al_2_(SO_4_)_3_ cations at pH 7. **Table S1**. Detailed properties of Zinc oxide nanoparticles used in this study. **Table S2**. Properties of the synthetic and natural waters used in the current study.
