# Supplementary material for: Interaction between Persistent Organic Pollutants and ZnO NPs in Synthetic and Natural Waters
Source: Nanomaterials (Basel). 2019 Mar 21;9(3):472. doi: 10.3390/nano9030472 (PMC6474098; doi:10.3390/nano9030472)
Supplement: Supplementary file 1 [file nanomaterials-09-00472-s001.pdf]

nanomaterials

Supplementary Material

Interaction between Persistent Organic Pollutants and ZnO NPs in Synthetic and Natural Waters

Rizwan Khan [1], Muhammad Ali Inam [1], Sarfaraz Khan [2], Du Ri Park [1] and Ick Tae Yeom [1,*]

[1] Graduate School of Water Resources, Sungkyunkwan University (SKKU) 2066, Suwon 16419, Korea; rizwankhan@skku.edu (R.K.); aliinam@skku.edu (M.A.I.); enfl8709@skku.edu (D.R.P.)

[2] Key Laboratory of the Three Gorges Reservoir Region Eco-Environment, State Ministry of Education, Chongqing University, Chongqing 400045, China; Sfk.jadoon@yahoo.com

* Correspondence: yeom@skku.edu; Tel.: +82-31-299-6699

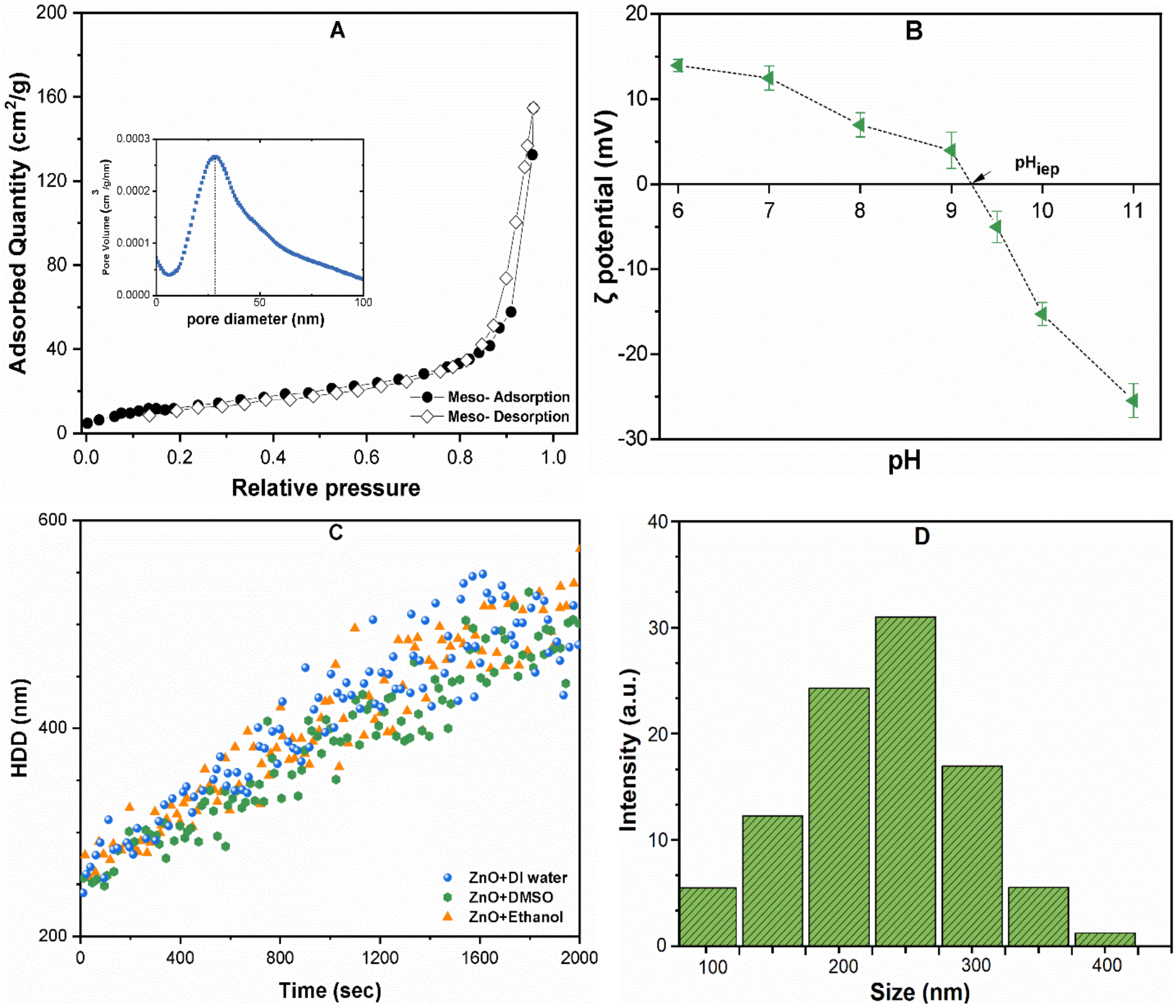

Figure S1. (**A**) Brunauer–Emmett–Teller (BET) analysis of zinc oxide nanoparticles (ZnO NPs) powder; (**B**) isoelectric point (IEP) of ZnO NPs in DI water; (**C**) aggregation kinetics of ZnO NPs in the absence and presence of the both solvents used for dissolving the polybrominated diphenyl ethers (PBDPEs); and (**D**) hydrodynamic size distribution of ZnO NPs in DI water after 30 min sonication

Table S1. Detailed properties of zinc oxide nanopatericles used in this study.

Nanomaterials Parameter	Unit	Technique	Value
Chemical formula			ZnO
Manufacturer-reported size[a]	nm	TEM	<50
Bulk density[a]	$g(cm^3)^{-1}$	–	5.60
Solubility			High
Isoelectric point (pH_{iep}, see Figure S1B)	–		9.2
Zeta potential in DI (pH=7)	mV	Malvern Zetasizer	+12 ± 5.1
HDD measured in DI (n=50)	nm	DLS	280 ± 35
Purity / moisture content	wt %	TGA/ICP-OES	96.52/1.85
Crystalline structure	–	XRD	Hexagonal
Shape			Polyhedral roughly round
Hamaker Constant	J[(b)]	–	1.9×10^{-20}
Net energy barrier in pure water (IS 5×10^{-6} M)	kT[(c)]	-	42.8

[a] Vendor reported

[b] [1]

[c] 1 KT=4.1142×10^{-21} J at 25 °C

ICP-OES: inductively coupled plasma optical emission spectroscopy

TGA: thermal gravimetric analysis

TEM: transmission electron microscopy

Table S2. Properties of the synthetic and natural waters used in the current study.

Parameter	Unit	Industrial Wastewater	Freshwater
pH *	–	7.54	6.90
IS	mML^{-1}	8.95	0.79
Conductivity*	μscm^{-1}	651	119
Na^{+}	mgL^{-1}	16.01	NM
K^{+}	mgL^{-1}	8.53	1.20
Cu	mgL^{-1}	0.36	NM
Fe	mgL^{-1}	ND	NM
Mg^{2+}	mgL^{-1}	28.15	3.49
As	mgL^{-1}	21.52	NM
Ca^{2+}	mgL^{-1}	16.11	1.50
Sb	mgL^{-1}	27.77	NM
Cl^{-}	mgL^{-1}	22.40	6.61
SO_4^{2-}	mgL^{-1}	10.52	NM
TOC	mgL^{-1}	25	10
HCO_3	mg CaCO3 L^{-1}	–	12
PO4	mgL^{-1}	ND	0.64

*Measured in lab

ND = Not detected

NM = Not measured

Results and Discussion

1.1. NPs Characterization

The Scherrer equation, D = (kλ/βhklcosθ), was used to determine the crystalline sizes of the ZnO NPs, where D is the crystalline size in nanometers (nm), λ is the wavelength of the radiation (1.54056 Å for CuKα radiation), k is a constant equal to 0.94, βhkl is the peak width at half-maximum intensity, and θ is the peak position. In this study (102) plane was chosen to calculate the crystalline size. The crystalline sizes of the ZnO NPs were observed to be 45 ± 2 nm, which is in accordance with the manufactured reported size (<50 nm).

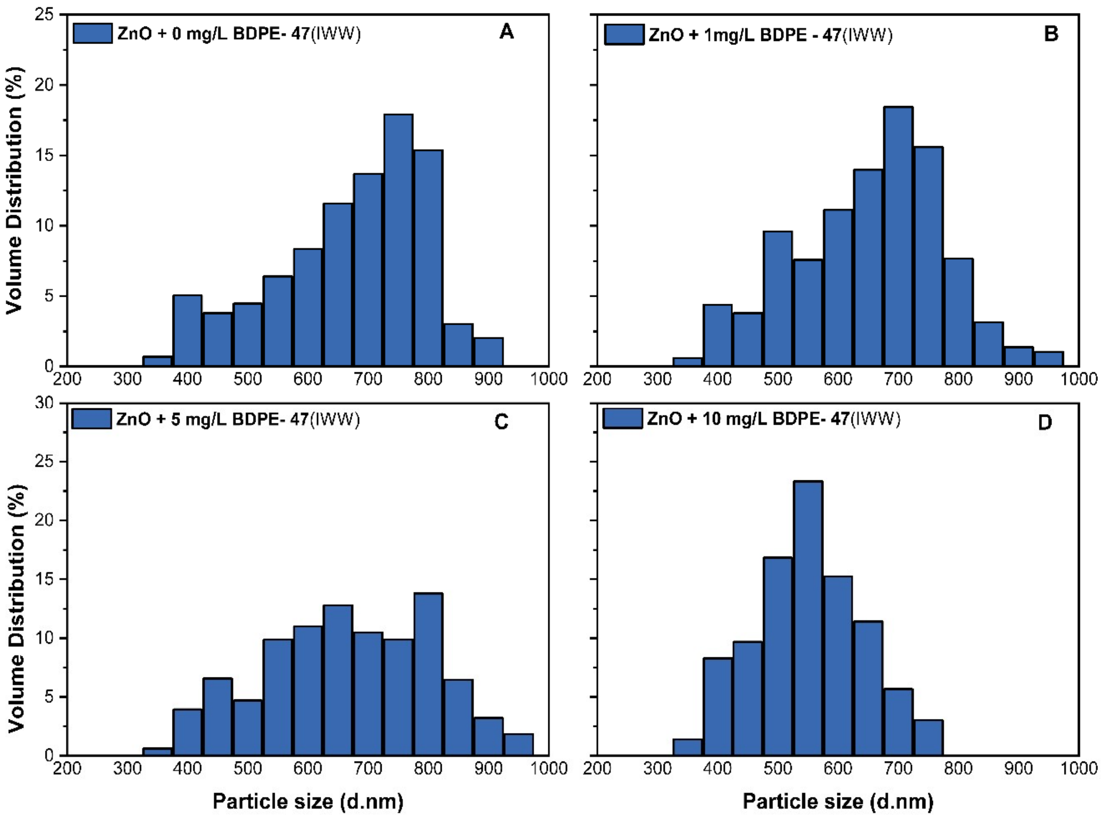

Figure S2. Volume distribution of ZnO NPs (10 mg/L) with different concentration of BDPE-47 showing (**A**) 0 mg/L; (**B**) 1 mg/L; (**C**) 5 mg/L; and (**D**) 10 mg/L in industrial wastewater (IWW).

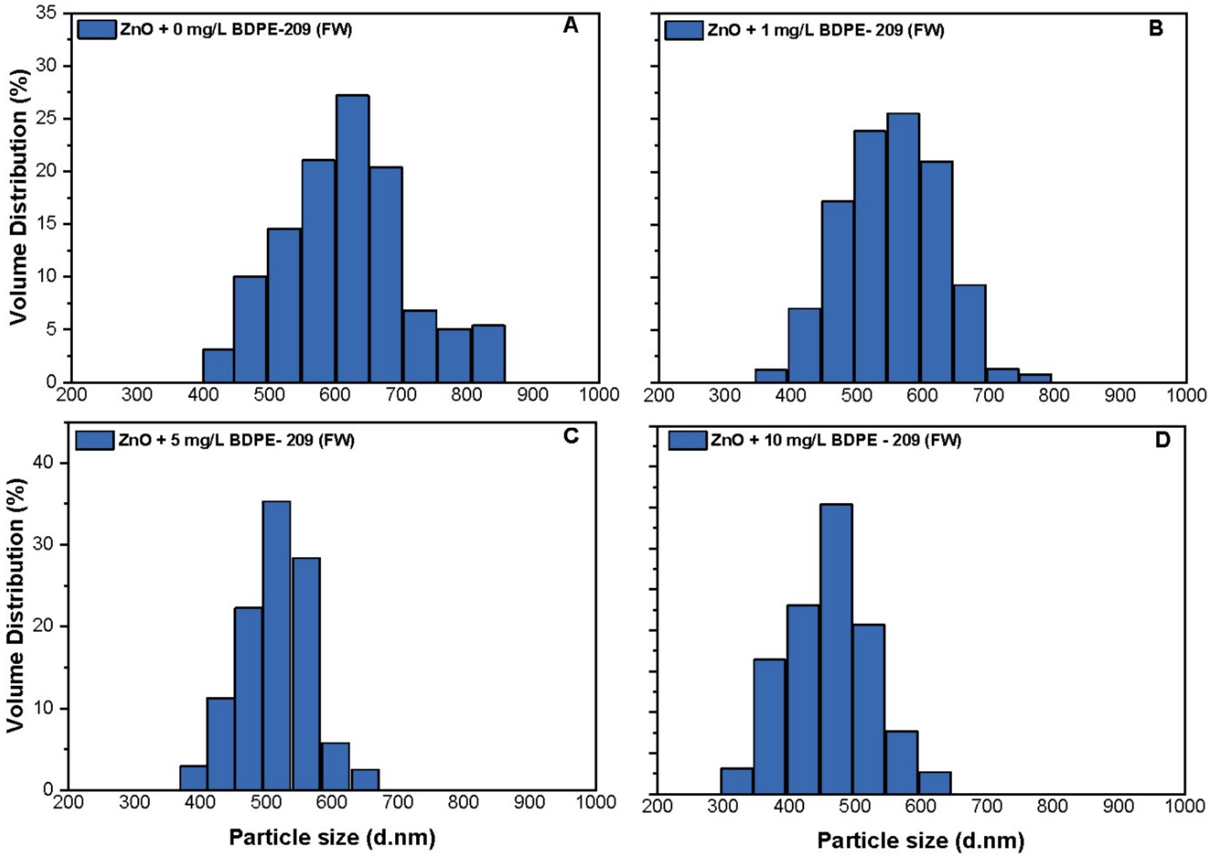

Figure S3. Volume distribution of ZnO NPs (10 mg/L) with different concentration of BDPE-209 showing (**A**) 0 mg/L ;(**B**) 1 mg/L; (**C**) 5 mg/L and; and (**D**) 10 mg/L in freshwater (FW).

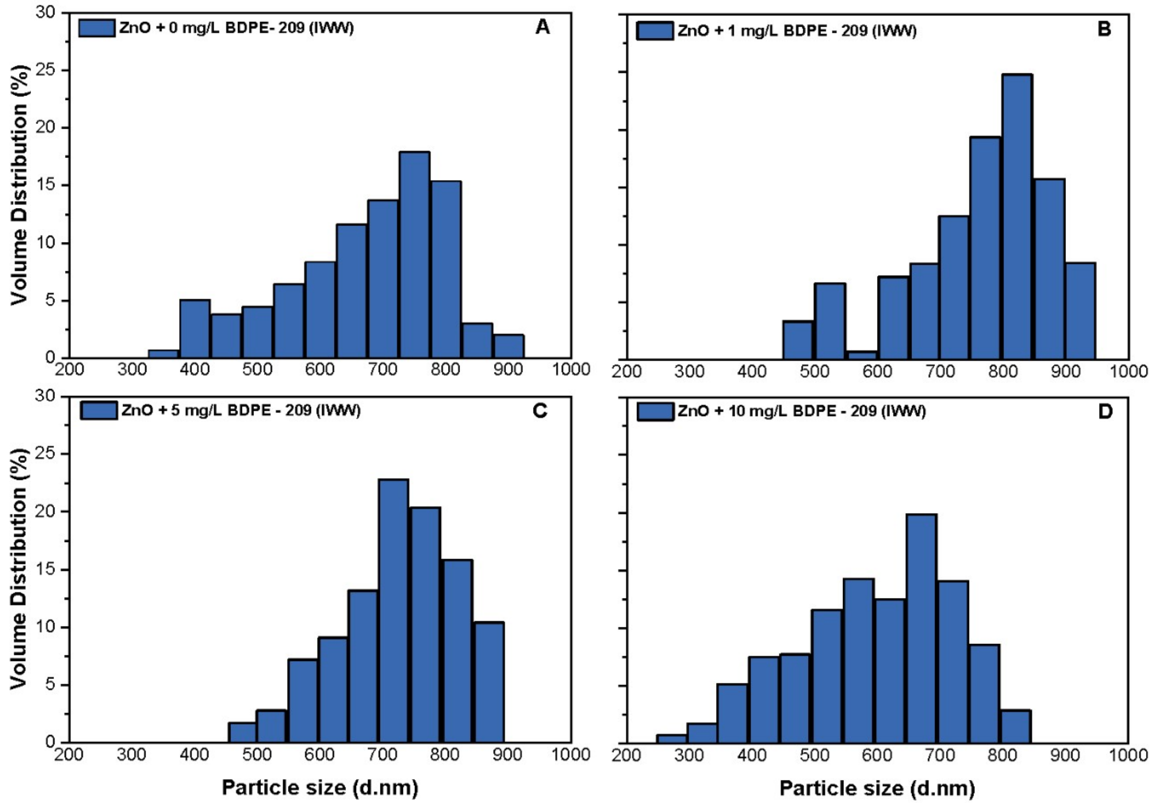

Figure S4. Volume distribution of ZnO NPs (10 mg/L) with different concentration of BDPE-209 showing (**A**) 0 mg/L ;(**B**) 1 mg/L; (**C**) 5 mg/L; and (**D**) 10 mg/L in IWW.

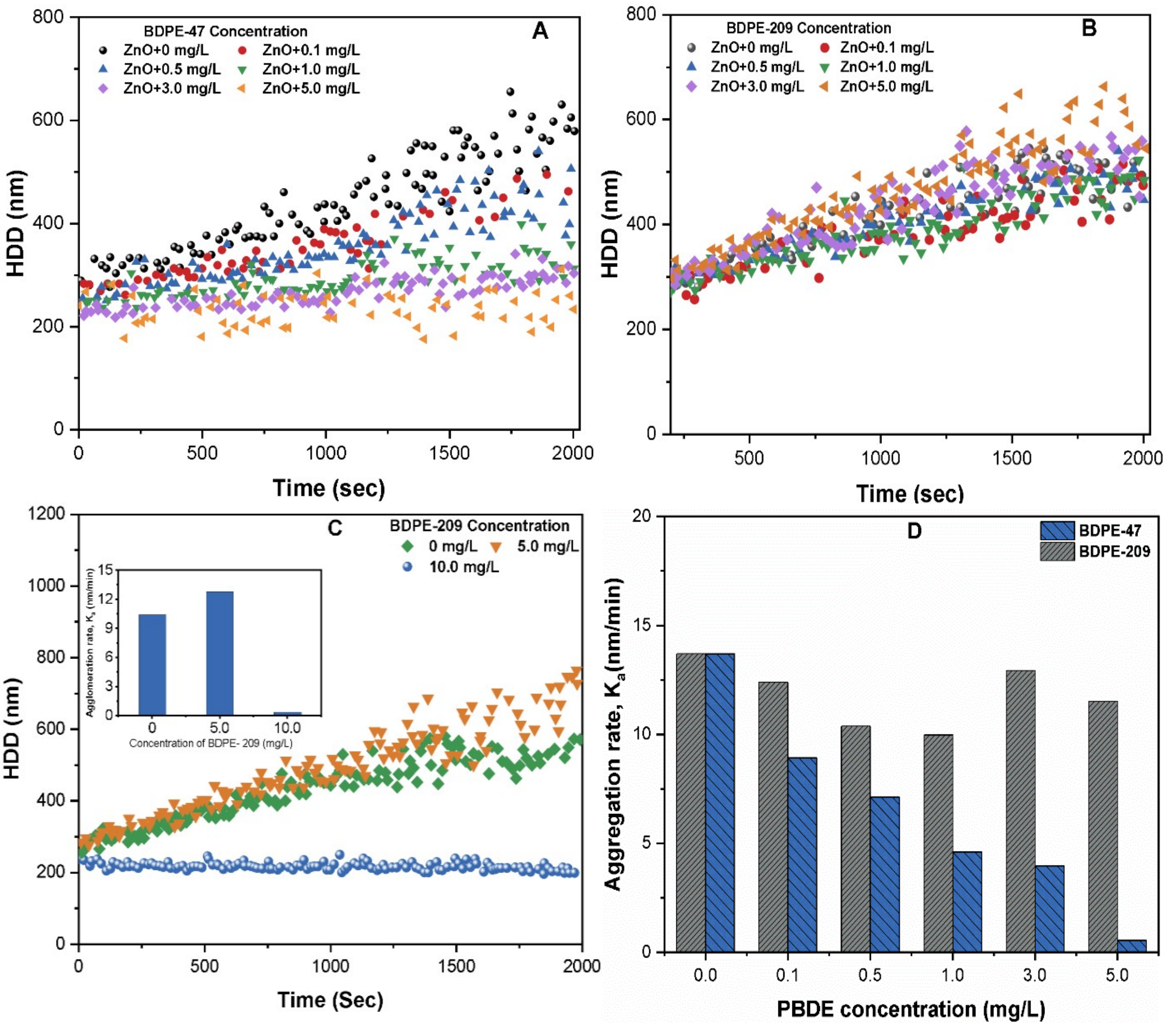

Figure S5. Effects of different concentration (0–5 mg/L) of (**A**) BDPE- 47 and (**B**) BDPE-209 on the aggregation kinetics of ZnO NPs (10 mg/L) in the presence of 20 mM KCl at pH 7; (**C**) aggregation kinetics of ZnO NPs in DI water containing 0, 5, and 10 mg/L BDPE-209, while inset showing aggregation rates at same concentrations; and (**D**) aggregation rates of ZnO NPs in at various concentration (0–5 mg/L) of BDPE- 47 and BDPE-209.

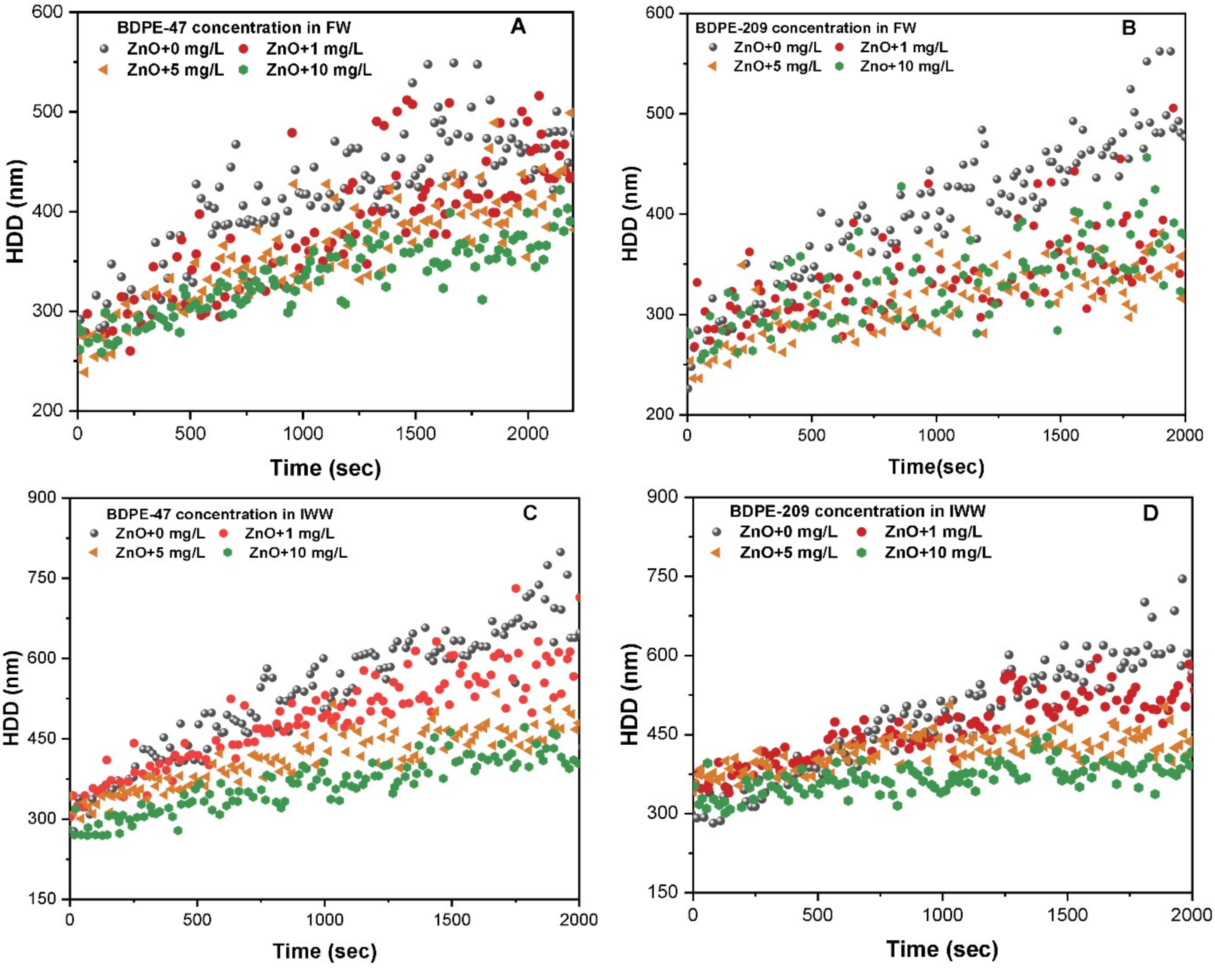

Figure S6. Effects of (**A**) BDPE- 47 and (**B**) BDPE-209 on the aggregation kinetics of ZnO NPs (10 mg/L) in FW, (**C**) BDPE -47, and (**D**) BDPE -209 on the aggregation kinetics of ZnO NPs in IWW.

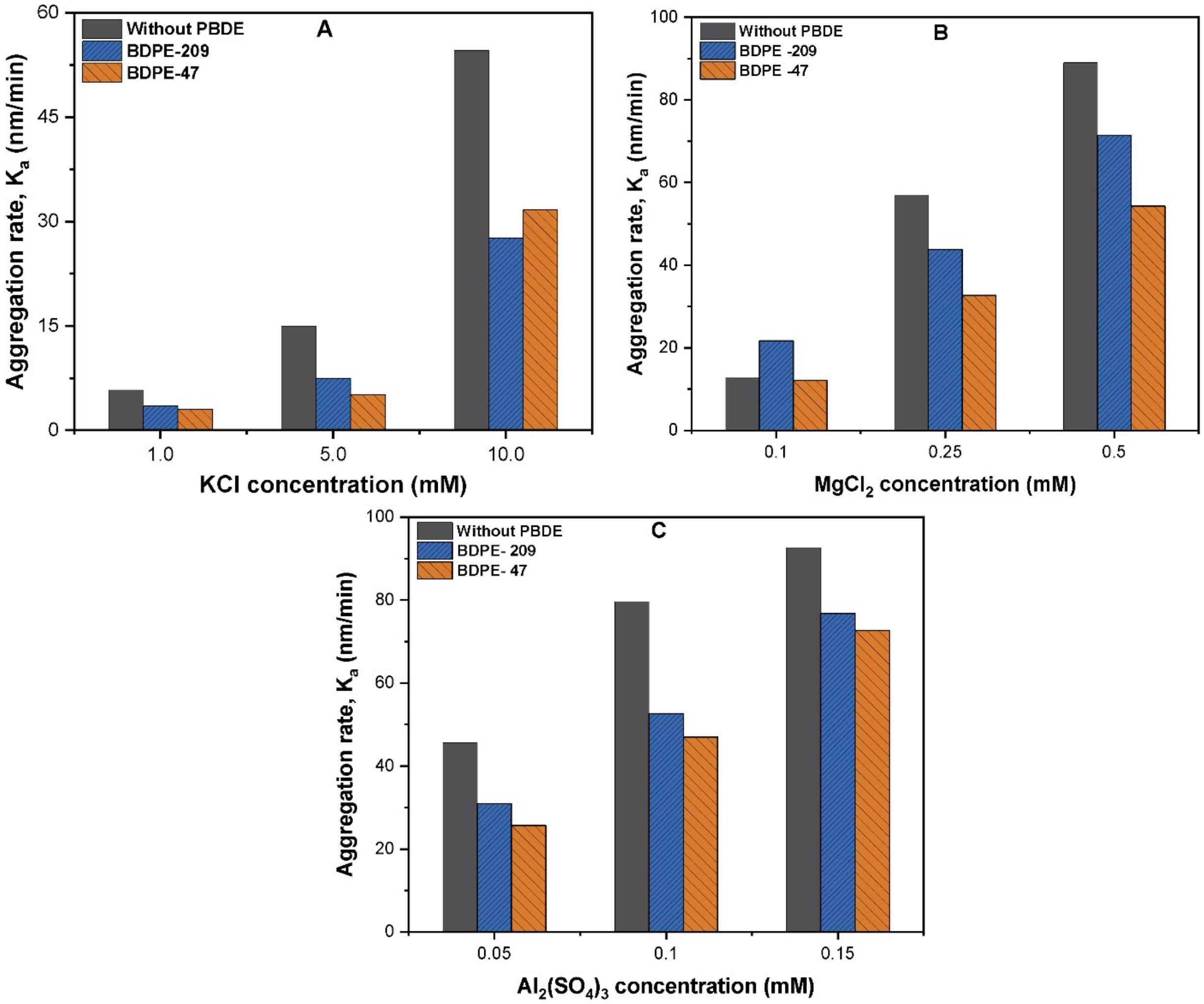

Figure S7. Aggregation rates of ZnO NPs (10 mg/L) in absence and presence (1 mg/L) of BDPE- 47 and BDPE -209 with (**A**) monovalent (KCl); (**B**) divalent ($MgCl_2$); and (**C**) trivalent $Al_2(SO_4)_3$ cations at pH 7.

References

1. Ackler, H. D.; French, R. H.; Chiang, Y. M. Comparisons of Hamaker constants for ceramic systems with intervening vacuum or water: From force laws and physical properties. *J. Colloid Interface Sci.* **1996**, *179*, 460–469, doi:10.1006/jcis.1996.0238.

© 2019 by the authors. Submitted for possible open access publication under the terms and conditions of the Creative Commons Attribution (CC BY) license (http://creativecommons.org/licenses/by/4.0/).
